# Occupational Exposure of Forest Workers to the Urticating Setae of the Pine Processionary Moth *Thaumetopoea pityocampa*

**DOI:** 10.3390/ijerph20064735

**Published:** 2023-03-08

**Authors:** Mario Olivieri, Enzo Ludovico, Andrea Battisti

**Affiliations:** 1 Unit of Occupational Medicine, Department of Diagnostics and Public Health, University of Verona, 37129 Verona, Italy; 2 Department DAFNAE, University of Padova, Legnaro, 35122 Padova, Italy

**Keywords:** insect, allergy, Pinus, Lepidoptera, Notodontidae

## Abstract

The larvae of the pine processionary moth are a threat to public health because they produce detachable setae that are about 200 µm long and 6 µm wide, reaching a total number of up to 1 million per mature individual. The setae are intended to be released to protect the larvae from predators but become a public health issue when in contact with humans and warm-blooded animals. Symptoms associated with the setae are typically urticaria and local swelling erythema, although edema of the skin, conjunctivitis or respiratory mucosa may occur. Occupational exposure concerns mainly forest workers but also farmers and gardeners. In the present study, we quantify the exposure to the setae of forest workers in a district of Northern Italy. The pine processionary moth represents a real case of occupational exposure as the urticating setae produced by the larvae caused symptoms in most forest workers directly in contact with the infested trees. In addition, the urticating setae were detected on the body of the chainsaw operators and in the surroundings of the felled trees during the operations. The non-exposed workers of the same agency did not report symptoms, with only one exception, likely linked to a non-occupational exposure. As the risk is not immediately perceived by the workers because direct contact with the larvae is unlikely, a campaign of information to workers and the general population living nearby infested forestry areas about the risk associated with airborne exposure is recommended. This becomes especially important in the areas of recent expansion of the insect, where people are inexperienced.

## 1. Introduction

**Figure 1:**
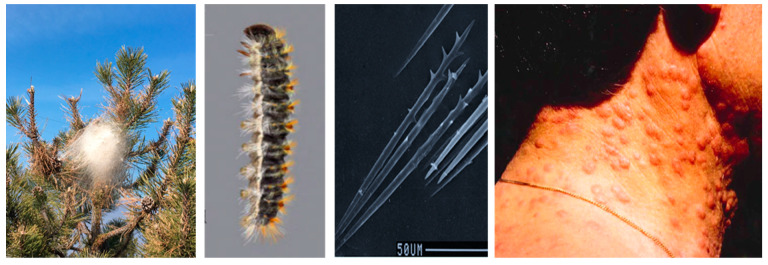
Pine tree (*Pinus nigra*) with a nest of the pine processionary moth, mature larva with the integument folds containing urticating setae on the back, urticating setae, pine processionary moth cutaneous reactions. Photographs by A. Battisti.

Processionary moths are insects carrying urticating setae as a larva and represent a threat to public health whenever they occur. The setae are intended to protect the larvae from predators, especially vertebrates such as birds [1]. When they come in contact with humans and warm-blooded animals, a series of health problems may arise, such as urticaria and local swelling erythema, conjunctivitis, or, due to inhalation in the respiratory airways, symptoms such as cough and shortness of breath [2]. Most of what we know about these organisms and the health problems they cause comes predominately from studies on one species, *Thaumetopoea pityocampa*, commonly known as the pine processionary moth (Figure 1). The larvae of this species live gregariously across the winter in conspicuous silk tents (nests) spun on the apical parts of the host plants (pines and cedars). The larvae produce detachable setae that are about 200 µm long and 6 µm wide on the abdominal tergites, reaching a total number of up to 1 million per mature individual [3]. Larvae can actively release the setae when threatened or when in contact with hard items, becoming a cause of aerial pollution [4]. Setae can also be deposited in the nests after the molting; as a result, nests become a source of setae [4].

People can be exposed to setae either through the air or by direct contact with larvae [1] or through contact with setae spread by the wind [5,6] (Figure 1). Once on the skin, the setae penetrate into the epidermis because of attrition caused by the hooks on their distal ends [7]. The eyes can also be affected, resulting in complex symptoms [8], while the respiratory system is rarely involved [9]. Reactions in humans are known to be strong and painful, especially in previously unexposed people [10]. In general, pine processionary moth cutaneous reactions are more common in rural areas where the main risk is related to exposure to this insect, and affected people show symptoms with various degrees of intensity [11]. Animals are also affected, especially domestic pets (cats, dogs), when they come into contact with the larvae (tongue necrosis) and grazing animals if setae are inadvertently ingested with the feed [12]. The reaction mechanism is associated with toxic proteins contained in the setae and released immediately after the seta penetration in the epidermis [13,14]. The delay between exposure and symptom appearance can extend to hours/days and can cause trouble in finding the correct diagnosis [15].

The proximity of pine and cedar trees to areas frequented by people and animals is a major factor of risk, and in cities within the geographic range of the pine processionary moth, specific control methods are taken [12]. The populations of the pine processionary moth fluctuate in time and space [16], and the density of the larvae is very important for assessing the risk of exposure to the urticating setae [12]. This is especially true for people professionally exposed to the risk, such as forest workers, farmers, and gardeners. A study conducted in Spain [9] showed that skin reactions caused by the larvae of the pine processionary moth were diagnosed as occupational immunologic urticaria in 30 patients belonging to several working categories such as pine-cone collectors/woodcutters, farmers/stockbreeders, construction workers, residential gardeners, and entomologists. In these patients, immediate hypersensitivity was demonstrated by performing skin prick and IgE-immunoblotting tests.

A comprehensive study comparing occupational exposure to the urticating setae of the pine processionary moth was not available in the literature. The aims of the present study are (a) to assess the occurrence of symptoms in exposed and non-exposed workers living in an area within the range of the pine processionary moth and (b) to measure exposure to setae during chainsaw felling of infested plants.

## 2. Materials and Methods

### 2.1. Occurrence of Symptoms in Exposed and Non-Exposed Workers

Eighty-eight workers of the Forest Service of Verona district in northern Italy (45.451710 N, 10.997716 E) were recruited as they were subjected to a medical examination for health surveillance by the Occupational Medicine Unit of the University Hospital of Verona. All workers were healthy and were suitable for the assigned task. Written informed consent was obtained from all patients, and the local ethical committee approved the study (ALPROC, 865CESC-Comitato Etico Sperimentazione Clinica). Each worker was asked to fill in a questionnaire about clinical manifestations from work that could be associated with exposure to trees infested by the larvae of the pine processionary moth. In particular, the questionnaire asked whether symptoms such as urticaria, conjunctivitis, cough, and shortness of breath occurred after the worker was involved in cutting trees carrying the nests of the pine processionary moth in the last 12 months. For the professionally non-exposed workers, the questions concerned the presence of the symptoms and whether the place of residence was close to areas infested by the moth.

### 2.2. Measure of Setae Density during Tree Felling

Forest workers (chainsaw operators) were selected during the ordinary activity of tree harvest and debranching in a *Pinus nigra* forest. The infestation level by the pine processionary moth level was moderate, with one nest per tree on average. In the period of the measures, the larvae were typically feeding at night-time and returning to the nest before sunrise [17]. The workers could be thus exposed to setae released by the larvae during the night feeding, e.g., if they were disturbed by a predator or shed by the nest during the tree felling and debranching operations. Each operator was assigned to an air pump (SKC Universal PCEXR4 Single Sample Pump Kit, SKC, 863 Valley View Road, Eighty Four, PA, USA) with a flow of 2 L/min, equipped with an IOM Multidust sampler using a PVC membrane filter of 5 µm. The samplers were located near the respiratory area of the operators in order to intercept the setae that could be inhaled through the nose and mouth. A total of ten sampling sessions were carried out on two operators in the morning (overcast sky, air temperature between 8 and 9 °C, relative humidity 80–100%) for a duration variable between 5 and 22 min. In addition, the samplers were also located at four distances (5, 10, 15, and 20 m) from the trees destined to felling during the operations, for a duration of 20–22 min for each session. At the end of the operations, Scotch^®^ Magic™ tape samples (1.5 cm × 4 cm) were applied to the skin of the forehead, neck, and wrist of the operators in order to detect the occurrence of the setae on uncovered skin. All tape samples were transferred to a microscope slide immediately after the application on the skin. Filters and slides were observed, and the setae were identified and counted under a microscope (magnification 100×) in the entomology laboratory of Padova University. At the end of the operations, the apical tree branches carrying the nests were removed and taken to the entomology laboratory of Padova University for assessing the larval instar and number. The nests were dissected in a hood with appropriate protection for the operators.

### 2.3. Statistical Analyses

The frequency of workers in the different categories was analyzed by χ^2^ test. Numbers of urticating setae detected in the environment and on the operators were analyzed by mean and standard deviation, and their abundance was compared by a *t*-test. Data were analyzed with the Microsoft Excel software, and a significance threshold of *p* < 0.05 was used.

## 3. Results

### 3.1. Occurrence of Symptoms in Exposed and Non-Exposed Workers

**Figure 2:**
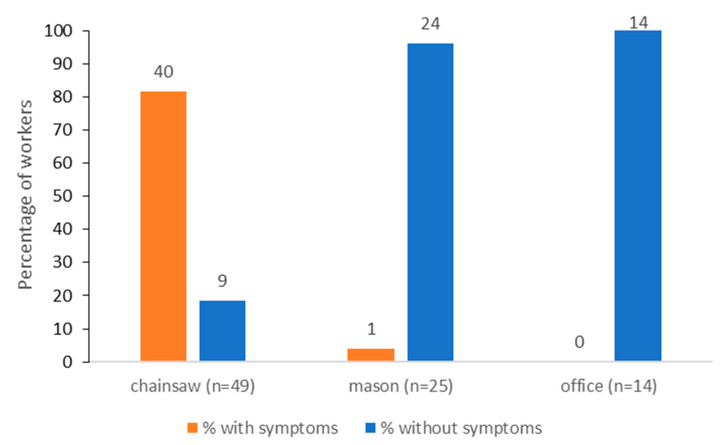
Frequency of symptoms associated with the exposure to the pine processionary moth in forestry workers assigned to different tasks.

The most common symptoms reported by the workers were itching of various types and extents, affecting mainly the exposed parts of the body. The workers resulted to belong to three groups according to specific tasks, with chainsaw operators being in significantly higher numbers than masons and office operators (*p* < 0.001) (Figure 2). The prevalence of symptoms was significantly higher among chainsaw operators (81.6%) than for masons (4%), while no symptoms were observed within office operators (*p* < 0.001) (Figure 2).

Chainsaw operators were generally familiar with the problem and reported a long history of exposure to the pine processionary moth, reporting that in some cases, forest operations were avoided or postponed in those stands heavily infested by the insect. The questionnaire indicated that tree felling and debranching was the activity causing the symptoms, even if no forestry worker reported direct contact with the insect. The parts of the body showing symptoms were mainly the neck and the wrists, and, in a few cases, eyelids. Non-exposed operators, such as office workers, reported no symptoms. Only 1 out of 25 masons living in a rural area with infested trees reported mild symptoms, not due to occupational exposure.

### 3.2. Measure Setae Density during the Exposure

During the tree felling operations carried out in the forest, a total of six trees were felled, each carrying one large nest on the apical part (Table 1). The larvae were in the final instar (L5) in all nests. The number of alive larvae in the nests spanned from 20 to 300, being roughly related to the nest size (Table 1).

The air pump filters detected setae either on the body of the operators or in the surroundings of the felled trees, although in different numbers (Table 2). Chainsaw operators collected many more setae than in the surroundings (*p* < 0.01), with an average exposure of 8.9 setae per hour of work, while 1.36 setae were trapped in the surroundings. The number of setae collected in the surroundings was too low to detect differences among the distances from the felled tree.

The tape samples taken from the skin of the chainsaw operators revealed the presence of very few urticating setae on all the body parts concerned (forehead, neck, and wrists). The number of setae found on the tape was too low to detect differences among the body parts.

## 4. Discussion

The pine processionary moth represents a real case of occupational exposure as the urticating setae produced by the larvae and sampled in this study caused symptoms in most forest workers exposed to infested trees. Chainsaw operators were highly susceptible, probably because the felling of the tree may dislodge the setae released by the larvae on the vegetation and from the nest. Reactions to the pine processionary moth do not give any specific clinical sign, so clinical diagnosis is generally based on the onset of skin reactions after exposure in the previous 24 h in an area with pine trees, usually in the winter months (January–April) [18]. In the last years, however, some authors [19,20] suggested entodermoscopy evaluation to confirm in vivo the diagnosis of skin reactions to the processionary moths, as it is applied to other agents affecting the skin, e.g., [21]. However, in another study where four human volunteers had setae applied to an area of 1 × 1 cm at the volar side of the forearm, the setae were detected by dermoscopy only at the magnification of 30×, while they were not visible at 10× magnification [18]. The use of tape applied to the skin after the exposure, followed by microscopic examination, appears to be a simple and effective method of setae detection to confirm occupational exposure, and it allows the preservation of the seta for further morphological identification [3]. Concerning exposure to airborne setae [22], operators acting close to the source of setae during chainsaw felling were clearly more exposed than persons at a distance of 15–20 m, in agreement with the higher frequency of chainsaw operators reporting symptoms. The data suggest that keeping a distance of 15–20 from the infested tree during chainsaw felling is enough for both safety and minimal exposure level to the setae.

The conditions in which the experiment was conducted were of mild infestation (one nest per tree on average), and the weather (low temperature and high humidity) was not favorable to setae dispersal [4,18]. Under conditions of higher infestation or with higher temperatures, it is recommended to avoid any tree-felling operation as there is a high potential for impacts on professionally exposed workers. Previous data on the impacts of the pine processionary moth on professionally exposed workers [9,23] reported symptoms of the same type as those registered in our study, which is the first to report the prevalence of the symptoms in groups of exposed and non-exposed workers. A previous history of exposure of the symptomatic workers to the setae could have amplified the appearance of symptoms because of a hypersensitive response [1].

Chainsaw workers were largely affected because of the proximity to the source of urticating setae, i.e., the branches where the larvae feed during the night and the nest where they rest during the day [17]. As long as the setae are thought to be a defense against vertebrate predators of the larvae [15], they are shed by the larvae in case of an attack, which is unlikely to happen during the tree harvest operations. The nest can be another source of setae, but the larvae are well separated from the outside by a thick layer of silk. These factors may explain the relatively low density of setae found on the operators and in the surrounding of the felled trees. Chainsaw operators are affected because they stay at the base of the tree, and the vibrations caused by the saw may induce the shedding of the setae from the canopy and from the nest. Setae are very light though and could be dispersed by air currents in many directions, as predicted by a model developed for the oak processionary moth [22]. In case of high population density or when the forest operations overlap with the movement of the larvae outside of the nest, the number of setae released could be much higher. The critical phase is when the mature larvae leave the nest on the tree to search for a pupation site in the ground, typically during the daytime in spring [24].

Occupational exposure of forest workers to urticating hairs produced by plant-feeding insects is known for a few cases where direct contact between the worker and the insect was demonstrated. This is the typical case of the pine caterpillar *Dendrolimus* spp. (Lasiocampidae) in China and Siberia, when the workers remove the cocoons by hand from the branches as part of pest control actions and come in contact with the modified setae embedded by the larvae in the cocoon [25]. In another case, the rubber extractors in Brazil may accidentally come in contact with the caterpillar hairs of *Premolis semirufa* (Erebidae) when they extract the latex [26]. In our study, the exposure of the chainsaw operators was indirect, as the larvae dispersed the setae in the habitat, and the workers did not report any direct contact with the larvae or the nests. The indirect exposure of workers to urticating setae produced by an arthropod has been shown for the theraphosid spiders of South America in a study center for tarantula in Brazil [27]. These spiders defend themselves against predators by releasing detachable urticating setae of various types [15].

The urticating setae in the air can be detected with air samplers as those used in the current study, generally used to detect airborne particles of medical importance. Alternatively, and in a more continuous way, the concentration of setae released into the air and their spatiotemporal aerial dispersal can be measured by spore or pollen traps, e.g., Hirst–Burkard traps. Due to the similar magnitude of sedimentation velocity and source location, aerial dispersion of pollen and setae was considered comparable in the case of the oak processionary moth [18]. Four trap types were used in the case of the pine processionary moth [28] and for the brown-tail moth *Euproctis chrysorrhoea* (Erebidae) in laboratory conditions [29]. The use of tape samples from the body of the operators was not that efficient in detecting the abundance of urticating setae, although it showed that setae could land on the body. The low efficiency is probably related to the limited skin area inspected. It would be useful to develop further the use of this method, especially in those cases where air pumps are not available or for a post-exposure diagnosis.

As chainsaw operators were shown to be the most exposed workers, it is recommended to survey the exposure to the urticating setae routinely when they operate in areas at risk. The skin parts that become exposed to the setae during the operations should be targeted for the detection of reactions and eventually urticating setae (tape samples). The possibility that setae released during the tree felling may be inhaled should also be considered, as well as the related reaction on the mucosa. Protection against those exposure risks is possible and consists of appropriate working gear, which is, however, limiting the efficiency of the work [2]. In addition, the gear should be disposed of after each use as the setae have a long persistence, and their removal is very difficult [2,7]. A practical solution to circumvent this problem is to postpone the forest operations to periods when the insect is not present, as peaks occur every 7-9 years [6]. A special situation may be encountered in areas of recent expansion of the pine processionary moth [30] or where the species has been accidentally introduced with plant trade [31]. Interestingly, the introduction of the oak processionary moth *Thaumetopoea processionea* in the UK has resulted in similar work-related hazards [32].

## 5. Conclusions

The pine processionary moth represents a special case of health risk for forest workers because the contact with the larvae is during the chainsaw felling of infested plants. In areas where the pine processionary moth is established, forest workers must be specifically trained to recognize the problem and adopt appropriate measures, mainly addressed to avoid contact with the setae. The strength of our study is to show for the first time the level of exposure of forest workers to the urticating setae of the pine processionary moth, indicating that chainsaw operators are by far the most exposed category. A limit that should be overcome in the future is represented by the difficulty in assessing the risk, as the air samplers are cumbersome to use, and the tape samples are not very efficient in detecting the presence of urticating setae. Campaigns of information specifically addressed to exposed workers and addressing the public are required to inform about the risk associated with the exposure of humans and domestic animals to the urticating setae of the pine processionary moth.

## Figures and Tables

**Table 1:** Traits of the pine processionary moth nests collected from the felled trees.

Tree/Nest	Diameter (cm)	Number of Larvae
1	12	20
2	20	80
3	15	30
4	18	20
5	30	300
6	45	150

**Table 2:** Number of urticating setae per minute found in the samples taken with air pumps during the tree-felling operations.

Item with Air Sampler	Mean Number of Setae/Liter/Minute	Standard Deviation
Chainsaw operator	0.148	0.140
Surroundings of felled tree (5–20 m)	0.023	0.025

## Data Availability

Data are available from the authors upon request.
